# Supplementary material for: A multi-dimensional measure of pro-environmental behavior for use across populations with varying levels of environmental involvement in the United States
Source: PLoS One. 2022 Oct 4;17(10):e0274083. doi: 10.1371/journal.pone.0274083 (PMC9531799; doi:10.1371/journal.pone.0274083)
Supplement: S1 Appendix — (DOCX) [file pone.0274083.s002.docx]

**Appendix A**

| Initial Pro-environmental Behavior Measures (adapted from [1-6]) |
| --- |
| Please rate yourself on how frequently you have participated in the following activities.  (0 = Never, 3 = Occasionally, 6 = As frequently as possible) |
| Recycled paper, plastic, and metal |
| Bought environmentally friendly and/or energy efficient products |
| Reused shopping bags |
| Walked or rode a bike when traveling short distances |
| Switched the lights off when I was the last person to leave the room |
| Reused or mended items rather than throwing them away |
| Composted food or yard and garden refuse |
| Avoided buying products with excessive packaging |
| Bought organic vegetables |
| Used rechargeable batteries |
| Waited until I had a full load before doing my laundry |
| Cut down on heating or air conditioning to limit energy use |
| Car-pooled when traveling to a destination |
| Limited time in the shower in order to conserve water |
| Made my yard or my land more desirable for wildlife |
| Participated (provided data) in a wildlife study |
| Volunteered to improve wildlife habitat in my community |
| Joined in community clean-up efforts |
| Grew vegetables and/or fruits at home |
| Talked to others in my community about environmental issues |
| Worked with others to address an environmental problem or issue |
| Participated as an active member in a local environmental group |
| Tried to convince friends to act responsibly towards the environment |
| Voted to support a policy/regulation that affects the local environment |
| Signed a petition about an environmental issue |
| Donated money to support local environmental protection |
| Wrote a letter in response to an environmental issue |

**References**

[1] Kaiser, F.G., & Wilson, M. (2000). Assessing people's general ecological behavior: A cross-cultural measure. *Journal of Applied Social Psychology, 30*(5), 952-978. DOI: https://doi.org/10.1111/j.1559-1816.2000.tb02505.x

[2] Vaske, J. J., & Kobrin, K. C. (2001). Place attachment and environmentally responsible behavior. *The Journal of Environmental Education*, *32*(4), 16-21. DOI: https://doi.org/10.1080/00958960109598658

[3] Markle, G.L. (2013). Pro-environmental behavior: does it matter how it’s measured? development and validation of the Pro-Environmental Behavior Scale (PEBS). *Human Ecology, 41*(6), 905-914. DOI: 10.1007/s10745-013-9614-8

[4] Larson, L. R., Stedman, R. C., Cooper, C. B., & Decker, D. J. (2015). Understanding the multi-dimensional structure of pro-environmental behavior. *Journal of Environmental Psychology*, *43*, 112-124. DOI: https://doi.org/10.1016/j.jenvp.2015.06.004

[5] Kaiser, F. G., Oerke, B., & Bogner, F. X. (2007). Behavior-based environmental attitude: Development of an instrument for adolescents. *Journal of Environmental Psychology*, *27*(3), 242-251. DOI: https://doi.org/10.1016/j.jenvp.2007.06.004

[6] Huddart-Kennedy, E., Beckley, T.M., McFarlane, B.L., & Nadeau, S. (2009). Rural-urban differences in environmental concern in Canada. *Rural Sociology, 74*(3), 309-329. DOI: https://doi.org/10.1526/003601109789037268
