# Supplementary material for: A multi-dimensional measure of pro-environmental behavior for use across populations with varying levels of environmental involvement in the United States
Source: PLoS One. 2022 Oct 4;17(10):e0274083. doi: 10.1371/journal.pone.0274083 (PMC9531799; doi:10.1371/journal.pone.0274083)
Supplement: S2 Appendix — (DOCX) [file pone.0274083.s003.docx]

**Appendix B**

For the pilot study, initial model fit was analyzed via ꭓ^2^, though it is recognized that this statistic is sensitive to large sample size [1]. To further assess model fit, alternative global fit indices were utilized: RMSEA ≤ 0.10 [1]; SRMR ≤ 0.08 [1]; and CFI ≥ 0.90 [2]. Bias-corrected confidence intervals were utilized in determining significance for factor loadings (95% confidence interval computed by 5000 bootstrap resamples) to minimize the likelihood of a Type 1 Error [3]. Factor loadings were deemed adequate if statistically significant and greater than 0.30 [4]. Cronbach’s Alpha was calculated for each sub-scale following the confirmatory factor analysis to determine sub-scale reliability [5]. Reliability was deemed to be adequate if the measure exceeded the 0.65 threshold established by [6]. All data analysis at this phase was conducted in IBM SPSS 26. The AMOS extension for SPSS was utilized for the confirmatory factor analysis.

This preliminary sample had a mean age of 46.56 years old with a minimum age of 18 and a maximum age of 94. Regarding race and ethnicity, which was not part of the initial quota sampling procedure, the sample was primarily white (85.6%). The reamining individuals identified as the following: Black or African American (5.2%), Asian or Pacific Islander (3.6%), Hispanic or Latino/Latina/LatinX (3.3%), Native American, American Indian, or Alaska Native (0.3%), and Other/Missing (1.9%). For gender, the sample self-identified as 52.10% female, 46.90% male, and 1.00% as non-binary. 26.3% of individuals reported having a household income of $100,000 or higher with the remaining sample fairly evenly spread below that amount at $10,000 increments. Of the 305 sampled, 12 individuals were missing data within the ERB measures and were thus excluded from analysis. This initial analysis indicated that 14 of the 27 items loaded well onto two latent constructs: Private Behaviors and Public Behaviors. A similar underlying structure of PEB has been supported by some previous research [7, 8], although it has not been operationalized into a validated, generalizable scale for a broader population. In examining the global fit indices of the confirmatory factor analysis, the ꭓ^2^ (ꭓ^2^=180.34, df=76, p<0.001) indicates poor model fit. Although, as mentioned previously, this statistic is sensitive to large sample sizes [1]. Alternative global fit indices indicate appropriate model fit: RMSEA=0.069; SRMR=0.048; CFI=0.950. Additionally, Cronbach’s Alpha values for Private Behaviors (0.86) and Public Behaviors (0.91) indicated appropriate reliability exceeding the 0.65 threshold established by [6].

Additional References

[1] Kline, R.B. (2016). *Principles and practice of structural equation modeling* (4^th^ ed.). Guilford Publications.

[2] Hu, L., & Bentler, P.M. (1998). Fit indices in covariance structure modeling: Sensitivity to underparameterized model misspecification. *Psychological Methods,* *3*(4), 424-453. DOI: https://doi.org/10.1037/1082-989X.3.4.424

[3] Byrne, B.M. (2001). *Structural equation modeling with AMOS: basic concepts, applications, and programming*. Lawrence Erlbaum Associates.

[4] Kline, P. (1994). *An easy guide to factor analysis.* Routledge.

[5] Cortina, J. M. (1993). What is coefficient alpha? An examination of theory and applications. *Journal of Applied Psychology*, *78*(1), 98. DOI: https://doi.org/10.1037/0021-9010.78.1.98

[6] Vaske, J. J. (2008). S*urvey research and analysis: Applications in parks, recreation and human dimensions.* Venture Publishing.

[7] Dono, J., Webb, J., & Richardson, B. (2010). The relationship between environmental activism, pro-environmental behaviour and social identity. *Journal of Environmental Psychology*, *30*(2), 178-186. DOI: https://doi.org/10.1016/j.jenvp.2009.11.006

[8] Sparks, A. C., Henderson, G. L., Sriram, S. K., & Smith, E. R. (2020). Measuring Environmental Values and Identity. *Society & Natural Resources*, 1-20. DOI: https://doi.org/10.1080/08941920.2020.1817644
