# Supplementary material for: A multi-dimensional measure of pro-environmental behavior for use across populations with varying levels of environmental involvement in the United States
Source: PLoS One. 2022 Oct 4;17(10):e0274083. doi: 10.1371/journal.pone.0274083 (PMC9531799; doi:10.1371/journal.pone.0274083)
Supplement: S3 Appendix — (DOCX) [file pone.0274083.s004.docx]

**Appendix C**

| Final PEB Scale | |
| --- | --- |
| Sub-Scale | **Please rate how frequently you have participated in the following activities in the past six months by selecting the appropriate point from the scale below** (Measured on a seven-point Likert scale ranging from 0 = “Never” to 6 = “As frequently as possible” |
| Private Behaviors | Bought environmentally friendly and/or energy efficient products |
|  | Walked or rode a bike when traveling short distances |
|  | Reused or mended items rather than throwing them away |
|  | Avoided buying products with excessive packaging |
|  | Bought organic vegetables |
|  | Minimized use of heating or air conditioning to limit energy use |
| Public Behaviors | Talked to others in your community about environmental issues |
|  | Worked with others to address an environmental problem or issue |
|  | Participated as an active member in a local environmental group |
|  | Signed a petition about an environmental issue |
|  | Donated money to support local environmental protection |
|  |  |
