# Supplementary material for: A multi-dimensional measure of pro-environmental behavior for use across populations with varying levels of environmental involvement in the United States
Source: PLoS One. 2022 Oct 4;17(10):e0274083. doi: 10.1371/journal.pone.0274083 (PMC9531799; doi:10.1371/journal.pone.0274083)
Supplement: Attachment — Submitted filename: PLOS_Reviewer_Response_Table_6-20-22.docx [file pone.0274083.s005.docx]

Dear Academic Editor and Reviewers,

Thank you for the careful revisions of our submitted manuscript. We appreciate the close and thoughtful eye that the manuscript has received from all three of you. The research team feels that the manuscript is greatly improved as a result of your insight and the corresponding revisions. Below you will find a table that specifically addresses each of your points and concerns about the manuscript. If a change was made in the manuscript’s writing, that is indicated in the table along with details about the revisions. We look forward to hearing your thoughts on the revised manuscript.

Best,

The Research Team

| **Academic Editor Comments** | **Response** |
| --- | --- |
| Please send a much more concrete manuscript | The manuscript has been revised extensively for clarity and detail. Additionally, extensive details on the theoretical nature of analyses have been added to the manuscript. It is our hope that this addresses the editors concerns about the manuscript. |
| I think that apart from the scale, by itself, this paper brings to the table a key issue in the measurement of PEBs. However, It seems to me you may be overestimating the pervasiveness of your scale. I need you to improve the discussion of how and when this scales is better than other approaches and acknowledge very clearly its limitations, most notably, vis a vis, behavioral measures | Theoretical details on the meaning of partial metric invariance have been added to the manuscript. The limitations on the comparability of the scale between the two groups is further emphasized in this expanded discussion. Additionally, in response to other reviewers and this comment, a section was added to the discussion on the tension this developed scale aims to balance: potentially limiting the breadth of behaviors measured in return for brevity and psychometric stability. In combination, the authors hope that this appropriately acknowledges the strengths and weaknesses of this novel scale in light of previously developed measures. |
| **Reviewer #1 Comments** | **Response** |
| Although the authors present several analyses and psychometric testing, to examine the scale’s reliability and validity, there exists some information to clarify:  -On page 18 you say: "a final confirmatory analysis was utilized at this stage (pilot) to indicate preliminary discriminant validity for the scale" (Brown, 2015). However, this is unclear because you do not explain how this analysis indicates discriminant validity (page 26). Moreover, the analysis is not presented in the results. So, the reader does not know the discriminant validity of the scale. | Clarification has been added to the referenced section describing how discriminant validity can be shown through a confirmatory factor analysis. We would also like to draw the readers attention to Table 1, which does display the results of the CFA from the pilot study. It was chosen to represent this table in the methods section, rather than the results section, as it primarily served as a way to determine which items to retain for the primary analysis presented in the remaining portions of the manuscript. |
| About the configural, metric, and scalar invariance:  -I recommend extending the explanation of the invariance metrics to understand better how they work.  -Some literature about invariance metrics refers to the need to run a Multigroup CFA even if the model fits well in each group. I think you should explain in detail if this analysis was done or not and why | Further information on the theoretical nature of each type of invariance has been added to the second-to-last paragraph in Section 4.4. Furthermore, clarification has been added to this paragraph that each type of measurement invariance was measured through a multi-group CFA that had sequentially greater restrictions between the group differences. |
| -On page 28 it says:  "When item loadings were released between groups for these four items, partial metric invariance was supported as changes in fit indices fell within all appropriate thresholds (ꭓ2=742.186, df=93, p<0.001; RMSEA=0.050; SRMR=0.0615; CFI=0.940). Releasing factor loadings for four items was considered appropriate as previous research has indicated that constraints should be maintained on at least half of the items for partial metric invariance to be confirmed (Steenkamp & Baumgartner, 1998; Vandenberg & Lance, 2000)".  I would suggest that at any point, before or after this paragraph, could be useful to know what partial invariance means, why it is a valid metric, and when to use it | Additional information has been added to this paragraph outlining what partial metric invariance means, both theoretically and practically for the scale. Limitations of this finding are also outlined. |
| -Although the analysis of scalar invariance failed, you do not present the implications of this result. In the discussion, it says the scale is reliable and valid, however, this is vague until you clarify the results of scalar invariance and its implications in the whole analysis of validity | In combination with the information added to address the previous point, implications on the developed scale being scalar noninvariant across the two groups was included in tandem with this information. The authors believe a description on scalar noninvariance pairs well with the previous information as it describes practical and theoretical limitations associated with analyses across the two groups of interest. |
| I wonder whether other analyses to test the validity of a scale could be useful to robust your paper, otherwise, could you explain why those analyses do not fit with the purpose of your study and, therefore, they were omitted? For example, discriminant validity and criterion-related validity (Kaiser & Biel, 2000; Kaiser, 1998) | As addressed in the previous point on discriminant validity and confirmatory factor analyses, our analysis did aim to display the discriminant validity of the developed scale. While there are certainly multiple other forms of validity that we did not explore in this manuscript, the analyses are already quite extensive, creating a fairly long piece of writing (>10,000 words). The authors feel adding additional analyses may further lengthen the manuscript unnecessarily. Going through each form of validity and describing why it was not utilized may also unnecessarily lengthen the manuscript. |
| Even though the purpose of the paper is "to develop a scale that measures a breadth of meaningful PEB, considering the psychological dimensions of these behaviors", the scale indeed does not include a breadth of PEB (e.g. water conservation and recycling behaviors are omitted). I suggest reporting the initial 27 items you use at the starting point to do the pilot study. I understand these items come from the previous literature about PEB. So, it could be useful to know the range of initial behaviors to understand what were the initial items dropped, and why some pro-environmental domains are missed in the whole analysis. I think it is confusing to present a scale about a breadth of meaningful PEB with important domains of private behaviors out. In the discussion, you say that your study suggests that previously established measures may over-specify the multidimensional nature of PEB. Probably, your scale may do an oversimplification because some domains are missing. | The initial 27 items utilized in the pilot study have been added as Appendix A to the manuscript. A section has been added to the last paragraph in the discussion section unpacking the tension we were aiming to balance in the developed scale. We may oversimplify PEB to a degree in our developed scale, but this is done in exchange for other benefits: increased psychometric stability and brevity. These pieces often exist in tension in scale development studies, and the authors hope that this additional information helps to clarify the balance that was sought. |
| There are some errors in the writing (duplicated words, typos, and wrong written words; e.g. pages 16 and 27) | The manuscript has been reviewed for typos, duplicated words, etc. These areas have been corrected where they have been found. |
| Page20: When you say: "Additionally, the 14 items measuring different aspects of PEB, outlined in Table 1", it is confusing the writing, and it seems imprecise to say different aspects. I suggest saying different PEB because you are measuring PEB, not aspects of them. | The language has been corrected here, indicating that we are measuring types of PEB rather than aspects of PEB. |
| I would like to know why the 6 months is a proper time-bound to consider the behaviors people performed? Why not 1 month, 3 months? | Clarification has been added to the manuscript on why this time bound was chosen. This clarification was added when the six month time bound was initially introduced. |
| Page 24: "For LNT, eigenvalues were 4.61 and 1.42 for the first two factors". -> These are not the same values that appear in Table 4. Please, match them. | Thank you for pointing out this typo. The analysis was re-run in R to confirm the correct values (4.63 and 1.41, those that were in Table 4). This has been corrected in the main body of the text. |
| Page 24: "An oblique rotation was utilized as the two latent factors were highly correlated with each other (0.61 for GEN and 0.55 for LNT)". -> Please, keep the same letters to identify the populations (GEN vs QUAL) because it could be confusing for the reader. | Yes, thank you for catching this typo! The language has been corrected in the referenced section. |
| In the Discussion, you say: "This study provides researchers and practitioners with a reliable and valid scale to measure general PEB in the United States across groups with different levels of involvement in  pro-environmental organizations or activities. Results suggested strong evidence for scale reliability and validity for both the LNT and QUAL groups". -> this affirmation seems a bit strong taking into account the limitations of the study. | The language was edited here to be a bit less strongly stated. Additionally, the limitations of only achieving partial metric invariance across the two groups was also added to this section to allow for a more responsible interpretation of study results. |
| In the Discussion (page 30) the sentence: "This is somewhat unsurprising given the significant role social norms have been found to play in influencing how PEB is enacted in various circumstances (Bamberg & Möser, 2007; Heberlein, 2012; Thomas & Sharp, 2013). Despite the influence social norms have been found to have on PEB, they have not been the primary factor determining the structure of a widely used PEB scale previously". -> it is confusing why the topic of social norms appears here, may you clarify? | Clarification has been added to the referenced section, indicating that public behaviors are generally social in nature. |
| In the Discussion (page 30) say: "Though this sub-scale structure has not been represented in previous studies, measuring general PEB through these two dimensions was determined to be psychometrically consistent across LNT and QUAL groups". -> this sentence is not clear because you say previously (in the same paragraph) that the bi-dimensional subscale structure is in line with previous research. | Clarification was added here to say that previous theoretical work on the nature of PEB has indicated a public/private split in behaviors, but this division has not been represented in the sub-scale structure of a previously developed scale. |
| In the Discussion (page 31) say: "For example, composting behavior was eventually removed from our scale due to its cross-loading between sub-scales amongst the QUAL group in the exploratory factor analysis,  indicating there may be some confounding factors influencing how this behavior is perceived and enacted. This aligns with findings by Huddart-Kennedy et al. (2009), suggesting that land stewardship behaviors are enacted less frequently in populations outside of rural areas". -> Here it is not clear whether you are talking about composting behavior or land stewardships behaviors, or ¿Are those behaviors being used interchangeably? | It is being asserted that composting is a single behavior falling underneath the broader umbrella of land stewardship behaviors (i.e., composting ultimately returns nutrients to the soil, primarily being used for gardening and farming). Clarifying language that composting is a specific behavior falling into the land stewardship category has been added to the reference section. |
| In the Conclusion, you mention a scale with 11 final items as a tool for future researchers, however, I could not see this final list of PEB in your supplementary material. I think it may be helpful to know it. | The final scale has been added to the manuscript as Appendix C. |
| The final paragraph in the Conclusion is a bit confusing. | The final paragraph has been revised for clarity. |
| **Reviewer #2 Comments** | **Response** |
| First, the authors start with a pilot and then use the scale developed in this pilot on two samples, and perform both exploratory and confirmatory factor analyses on these two samples. It would have been nice to see actually a new sample to confirm the structure of the scale, however, this could also be done in a follow-up study. I would, however, like to see at least a discussion of this - why didn´t the authors go out-of-sample for the confirmatory factor analysis? | A paragraph has been added to the limitations section addressing this comment. While an additional out-of-sample confirmatory factor analysis would provide additional rigor for the study, the authors feel that the additional rigor provided by adding a fourth population to this study would not be justified given the resources it would take to carry out this additional sampling and analysis. Scale properties are supported by various analyses run across three separate populations. |
| Secondly, the paper could be shortened considerably. The authors repeat their main points quite a lot, and by that prolong the text unneccesarily. Focusing the paper would increase its value and probably also the reception it will get by readers. | The manuscript has been reviewed for length and clarity. Various sentences have been trimmed at different points to reduce word count slightly. The authors generally feel arguments are made at appropriate times and when they are necessary in the manuscript. |
| I am a bit surprised that the authors start with the argumentation that context very strongly influences PEB, but then go on to develop a scale that should be entirely consistent across two very distinct populations. Of course, having a very short scale is nice, and having only dimensions as well, in the sense of "ockham´s razor". However, I would like to see a more thorough discussion whether this does not under- or overestimate PEB in some subpopulations whose PEB is not captured in the items of the scale? | Information has been added to the end of the discussion section relevant to this point. The authors discuss that they aimed to balance various scale aspects in developing this new measure. A level of oversimplification may be present in this scale in return for two primary benefits: psychometric stability and brevity. While we recognize there are limitations to the scale we have developed, we feel it also provides certain benefits that have not been provided by previous PEB measures. |
| Finally, the authors mention that this study is part of an alternate study for which data were being collected. While I think publishing the scale-development of a larger project has merits, it would be important to have more information on the overall study. | Additional information on the broader study that this scale development project was part of has been added to the end of section 4.3. |
| A very minor point - in the description of the factor analyses, the authors mention that in the PAF they use a cutoff-value of 0.32, and later one of 0.30. Is there any argument for that difference? | In reviewing the manuscript, the authors are having trouble identifying the discrepancy noted here. The cutoff for loadings for PAF (as a form of EFA) was 0.32. The adequate factor loading for confirmatory factor analyses was 0.30. These are two separate statistical analyses, so cutoff values may differ between the two statistical procedures. Citations are provided for the two different cutoff values. |
